# Novel Harmicines with Improved Potency against *Plasmodium*

**DOI:** 10.3390/molecules25194376

**Published:** 2020-09-23

**Authors:** Marina Marinović, Ivana Perković, Diana Fontinha, Miguel Prudêncio, Jana Held, Lais Pessanha de Carvalho, Tana Tandarić, Robert Vianello, Branka Zorc, Zrinka Rajić

**Affiliations:** 1 Faculty of Pharmacy and Biochemistry, University of Zagreb, A. Kovačića 1, HR-10 000 Zagreb, Croatia; mmarinovic@pharma.hr (M.M.); iperkovic@pharma.hr (I.P.); bzorc@pharma.hr (B.Z.); 2 Instituto de Medicina Molecular, Faculdade de Medicina, Universidade de Lisboa, Av. Prof. Egas Moniz, 1649-028 Lisboa, Portugal; dfontinha@medicina.ulisboa.pt (D.F.); mprudencio@medicina.ulisboa.pt (M.P.); 3 Institute of Tropical Medicine, University of Tübingen, Wilhelmstraße 27, 72074 Tübingen, Germany; janaheld@hotmail.de (J.H.); lais_pessanha@hotmail.com (L.P.d.C.); 4 Computational Organic Chemistry and Biochemistry Group, Rudjer Bošković Institute, Bijenička cesta 54, HR-10 000 Zagreb, Croatia; tana.tandaric@irb.hr (T.T.); Robert.vianello@irb.hr (R.V.)

**Keywords:** harmine, cinnamic acid, amide, antiplasmodial activity, *Pf*Hsp90, molecular dynamics simulations, *P. berghei*, P. falciparum

## Abstract

Harmicines represent hybrid compounds composed of β-carboline alkaloid harmine and cinnamic acid derivatives (CADs). In this paper we report the synthesis of amide-type harmicines and the evaluation of their biological activity. *N*-harmicines **5a**–**f** and *O*-harmicines **6a**–**h** were prepared by a straightforward synthetic procedure, from harmine-based amines and CADs using standard coupling conditions, 1-[bis(dimethylamino)methylene]-1*H*-1,2,3-triazolo [4,5-*b*]pyridinium 3-oxid hexafluorophosphate (HATU) and *N*,*N*-diisopropylethylamine (DIEA). Amide-type harmicines exerted remarkable activity against the erythrocytic stage of *P. falciparum*, in low submicromolar concentrations, which was significantly more pronounced compared to their antiplasmodial activity against the hepatic stages of *P. berghei*. Furthermore, a cytotoxicity assay against the human liver hepatocellular carcinoma cell line (HepG2) revealed favorable selectivity indices of the most active harmicines. Molecular dynamics simulations demonstrated the binding of ligands within the ATP binding site of *Pf*Hsp90, while the calculated binding free energies confirmed higher activity of *N*-harmicines **5** over their *O*-substituted analogues **6**. Amino acids predominantly affecting the binding were identified, which provided guidelines for the further derivatization of the harmine framework towards more efficient agents.

## 1. Introduction

Malaria is a life-threatening parasitic disease that kills over 400,000 people each year, mainly children under 5 years of age in sub-Saharan Africa [1]. Five species of *Plasmodium* cause malaria in humans, and the most severe form of the disease is caused by *P. falciparum*, which is also the most prevalent malaria parasite [2]. The complex life cycle of *Plasmodium* includes two hosts, *Anopheles* mosquitoes and mammals. In the latter, a clinically silent phase of hepatic infection obligatorily precedes a subsequent erythrocytic infection stage, responsible for the symptoms of malaria [3]. Since 2001, when the WHO recommended the use of artemisinin-based combination therapies (ACTs) for treating *P. falciparum* malaria, ACTs have become the mainstay of malaria therapy due to their high efficacy, safety and lack of serious side effects [4]. Although malaria-caused deaths continuously drop and several countries report either none or under 100 new cases each year, the rise of drug-resistant parasites has slowed down that progress, especially in Asia. Failure rates of *P. falciparum* treatments to the first-line ACTs were found to be above 10% in the Southeast Asia Region, and were as high as 93% in Thailand [1]. Despite numerous efforts to develop an effective vaccine against malaria, it still remains out of reach [5,6,7]. Taken together, the rising number of drug-resistant parasite strains and the unavailability of an effective vaccine exemplify the need to look for novel antimalarial agents.

**Figure 1:**
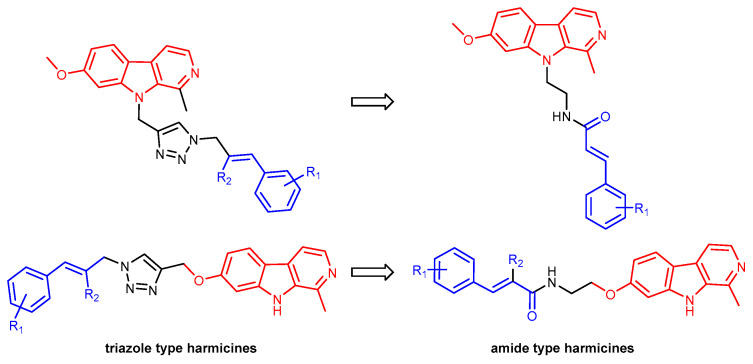
Triazole and amide-type harmicines (harmine is marked in red, while the cinnamic acid derivative (CAD) scaffold is marked in blue).

Hybrid drugs, compounds with at least two distinct pharmacophores, represent one of the possible strategies for finding novel biologically active molecules [8,9]. As previously shown by us and others, the preparation of hybrids based on known antimalarial agents and cinnamic acid (*trans*-3-phenyl-2-propenoic acid) or its derivatives (CAD) resulted in an increase in the former’s in vitro antiplasmodial activity [10,11,12,13,14,15]. In our previous publication, we reported the synthesis and biological activity of triazole-type harmicines, hybrids based on the alkaloid harmine and CAD, linked via 1*H*-1,2,3-triazole (Figure 1) [15]. Harmine, a natural β-carboline alkaloid, was chosen as a CAD partner due to its known in vitro and in vivo antiplasmodial activity [16,17]. We showed that triazole-type harmicines exert a significant activity against both erythrocytic and hepatic stages of *Plasmodium* infection. In addition, molecular dynamics (MD) simulations confirmed the binding of the most active compound within the ATP binding site of *P. falciparum* heat shock protein 90 (*Pf*Hsp90) [15], which is essential for the parasite’s development, and may play a major role in drug resistance [16,17,18,19,20].

Since our earlier MD simulations did not identify the triazole ring as being crucial for binding to *Pf*Hsp90 [15], we decided to simplify the linker between two main fragments, harmine and CAD. To this end, triazole was replaced with its bioisostere, an amide bond, resulting in novel, amide-type harmicines. Here, we report the synthesis of amide-type harmicines, their activity against both erythrocytic and hepatic stages of *Plasmodium* infection, and their cytotoxicity. We also discuss how binding to *Pf*Hsp90 affects their activity.

## 2. Results and Discussion

### 2.1. Chemistry

As a continuation of our work on hybrids based on harmine and CADs, we decided to focus on amide-type harmicines **5** and **6**. Before synthesis, we evaluated the drug-like properties of the planned compounds, by calculating relevant physico-chemical parameters included in the Lipinski’s rule of five and Gelovani’s rules for small molecules, using Chemicalize.org software [21]. To our delight, all compounds were in complete agreement with both sets of rules (Table S1).

**Scheme 1:**
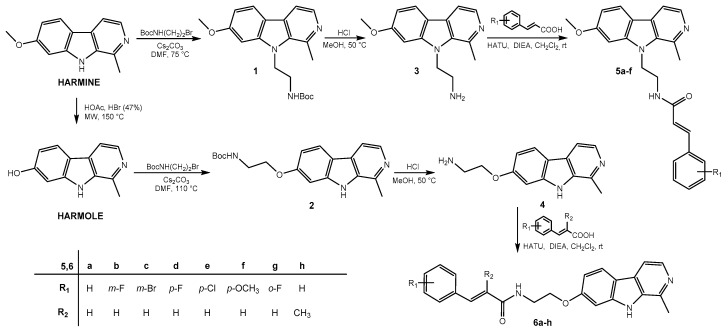
Synthesis of harmicines **5** and **6**.

The chosen synthetic pathway towards novel harmicines involved three reaction steps, depicted in Scheme 1. In the first step, harmine and harmole were alkylated with 2-(Boc-amino)ethyl bromide to yield *N*- and *O*-alkylated compounds **1** and **2**, respectively. The alkylation of harmine proceeded smoothly with four equivalents of 2-(Boc-amino)ethyl bromide in the presence of four equivalents of Cs_2_CO_3_, at 75 °C for 24 h, whereas employing the same reaction conditions for the alkylation of harmole resulted in the formation of an *N*,*O*-bis alkylated product. A careful optimization of the reaction conditions allowed the selective *O*-alkylation of harmole. Only traces of the *N*,*O*-bis alkylated product were obtained when 2.6 equivalents of the alkylating agent and 1.35 equivalents of Cs_2_CO_3_ were employed at 110 °C for 4 h.

The Boc-protecting group was efficiently removed under acidic conditions, to attain primary amines **3** and **4**. In the final reaction step, the coupling between amines **3** or **4** and CADs was accomplished by using standard coupling conditions, 1-[bis(dimethylamino)methylene]-1*H*-1,2,3-triazolo [4,5-*b*]pyridinium 3-oxid hexafluorophosphate (HATU) and *N*,*N*-diisopropylethylamine (DIEA) in dichloromethane (DCM). The employed CADs were chosen according to the results of biological assays obtained earlier [15]. Coupling reactions were performed at room temperature, reaction times were short (1–2 h), and synthetic yields were moderate to high (47–77%). Purification of the crude products by column chromatography and/or crystallization was straightforward. Structures of the final products were confirmed by standard spectroscopic/spectrometric methods (^1^H and ^13^C NMR, IR, MS), while their purity was assessed by elemental analyses. The data obtained are provided in short in the Materials and Methods section, and in detail in the Supplementary Information.

### 2.2. Biological Evaluations

#### 2.2.1. Antiplasmodial Activity

After the successful preparation and characterization of the title compounds, we sought to assess their in vitro activities against the erythrocytic and hepatic stages of *Plasmodium* infection and to investigate whether they differ from those activities of the triazole-type harmicines reported earlier [15].

##### In Vitro Activity against *P. falciparum* Erythrocytic Stage

The in vitro antiplasmodial activity of harmicines against *P. falciparum* erythrocytic stage was assessed employing both the chloroquine-sensitive (*Pf*3D7) and the chloroquine-resistant (*Pf*Dd2) strains, as previously described [22,23,24] (Table 1). Chloroquine and harmine were included as positive controls in these assays, and the activity of amide-type harmicines was compared with that of the previously prepared triazole-type harmicines [15]. The conjugation of harmine to CADs yielded compounds with significantly higher antiplasmodial activity against *P. falciparum* blood stages than the parent compound, harmine. Remarkably, the activities were in the low submicromolar (*Pf*3D7) and submicromolar (*Pf*Dd2) concentrations, which is an activity one order of magnitude stronger than that exerted by the triazole-type harmicines. To our surprise, and in contrast with the data obtained for the triazole-type harmicines, analysis of the IC_50_ (concentration of the tested compound necessary for 50% growth inhibition) values for homologues *N*- and *O*-harmicines showed that the antiplasmodial activity of *N*-harmicines **5** is significantly higher than that of *O*-harmicines **6** against both strains, except in the case of compounds **5f** and **6f** (*Pf*3D7), as well as **5c** and **6c** (*Pf*Dd2). 

We further compared the IC_50_ values within both series and found that the effects of the substituents in the cinnamic scaffold on the antiplasmodial activity were more pronounced for the *Pf*3D7 strain. As shown in Table 1, *N*-harmicines bearing halogens either in the *p*- or the *m*-positions (**5b**–**e**) exhibited significantly higher activities than unsubstituted and *p*-methoxy substituted compounds. In the *O*-harmicines **6** series, a substitution in the *p*-position was preferred, regardless of the type of substituent. Interestingly, the substitution of hydrogen with its isostere fluorine in the *p*-position yielded the most active compound among *O*-harmicines (**6d**), while the same substitution in the *m*-position resulted in decreased activity. The replacement of hydrogen with fluorine in the *o*-position had no significant influence on the antiplasmodial activity. Conversely, the substitution of the hydrogen with a methyl group in the α-position yielded compound **6h**, with at least one order of magnitude weaker antiplasmodial activity than other *O*-harmicines. The influence of lipophilicity was estimated by the comparison of IC_50_ and calculated log *P* values (Table S1). More lipophilic *N*-harmicines exerted more pronounced activity, while *O*-harmicines did not follow the same pattern. The most active compound against both strains was *N*-harmicine **5e** (IC_50_(*Pf*3D7) = 0.04 μM, IC_50_(*Pf*Dd2) = 0.17 μM).

##### In Vitro Activity against *P. berghei* Hepatic Stages

**Figure 2:**
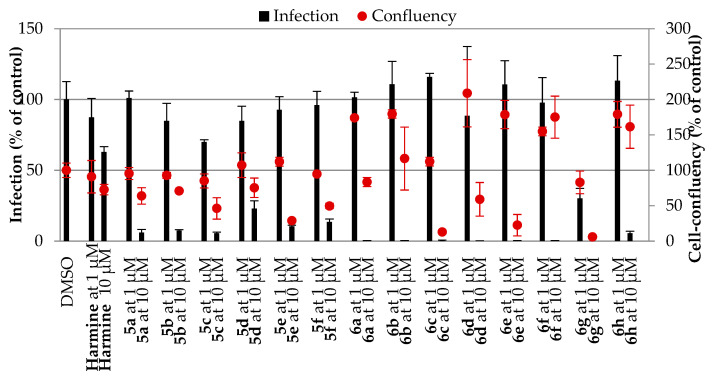
In vitro activity against *P. berghei* liver stages of harmicines **5a**–**f** and **6a**–**h** at 1 and 10 μM concentrations. Total parasite load (infection scale, bars) and cell viability (cell confluency scale, dots) are shown. Results were normalized to the negative control, DMSO, and are represented as mean ± SD, *n* = 1.

The in vitro activity of harmicines against the hepatic stages of *Plasmodium* infection was evaluated by employing rodent *P. berghei* parasites and a human hepatoma cell line (Huh7), as previously described [25,26]. Each compound was assessed at 1 and 10 μM (Figure 2), with harmine and DMSO serving as positive and negative controls, respectively. Concomitantly, the compounds’ cytotoxicity to Huh7 cells was assessed by cell confluency measurement. The results obtained show that amide-type harmicines do not exert significant activity against the hepatic stages of *P. berghei* infection. The compounds’ activity at 1 μM is similar to that of the parent compound harmine, but they are significantly more active than the latter at 10 μM. The marked cytotoxicity observed to Huh7 cells, even at 1 μM, precluded the determination of IC_50_ values for the compounds’ hepatic stages antiplasmodial activity.

#### 2.2.2. Cytotoxicity Assay

A cytotoxicity assay was performed using the neutral red assay, as described previously, against human liver hepatocellular carcinoma cell line (HepG2) [27] (Table 1). For the safety assessment, a selectivity index (SI) for each compound was calculated as the fractional ratio between the IC_50_ values for HepG2 and *P. falciparum Pf*3D7 strain (Table 2). In general, *N*-harmicines **5** are less cytotoxic than *O*-harmicines **6**, which is in agreement with the results obtained for triazole-type harmicines. Furthermore, all *N*-harmicines, but only two *O*-harmicines, **6d** and **6f**, have more favorable SIs than the parent compound harmine (SI = 30). A close inspection of the IC_50_ values within the series of *N*-harmicines revealed that substitution of hydrogen in the cinnamic moiety by F, Cl or Br led to a significant increase in cytotoxicity (H < F < Cl ≈ Br). *O*-harmicines showed similar levels of cytotoxicity, independently of the cinnamic substituent. Compound **6h**, with a methyl group in the α-position in the CAD region, was the only exception, as it was significantly less toxic than the other *O*-harmicines. Compound **5b**, with a *m*-fluorine substituent in the cinnamic scaffold, exerted the highest selectivity (SI = 773), followed by **5a**, an amide with unsubstituted cinnamic acid (SI = 715). Two other compounds, namely **5f** and **5d**, also showed pronounced selectivity (SIs 287 and 233, respectively), whereas **6h** and **6b** displayed low selectivity (SIs 9 and 5, respectively).

### 2.3. Molecular Dynamics Simulations

Computational analysis was performed to gain an insight into the binding of a representative set of ligands, involving both *N*-substituted (**5a**, **5d** and **5e**) and *O*-substituted derivatives (**6a**, **6d** and **6e**), and to interpret their observed affinities towards *Pf*Hsp90. To this end, MD simulations were employed to obtain the binding poses and the accompanying binding free energies, as well as to identify residues governing the binding. These results were compared with those previously obtained for the parent harmine [15]. 

We calculated the selected compounds’ binding free energies (Δ*G*_BIND_), as well as their decomposition into contributions from individual residues (Table 2). The specific residues considered for the analysis include those responsible for the binding of **5e** within the ATP binding pocket (Asn37, Asp79, Arg98, Phe124) [28,29], and all those with contributions higher than −0.50 and lower than 0.03 kcal mol^−1^, leading to the identification of the residues that are the most and the least responsible for the binding of **5e** to *Pf*Hsp90.

Our data reveal that all evaluated compounds are associated with negative Δ*G*_BIND_, in agreement with their high antiplasmodial activity. In addition, a very good agreement between calculated data and experimentally measured activities is observed. Firstly, with the highest binding free energy of Δ*G*_BIND_ = −40.9 kcal mol^−1^, computations clearly predict **5e** to have the highest activity, which was confirmed experimentally. It is worth noting that this value exceeds that of an analogous triazole-based *N*-substituted derivative studied earlier [15], where the computed value was −39.0 kcal mol^−1^, being strongly in line with a general tendency of a higher activity for the amide-based derivatives examined here. Moreover, the calculated difference of −1.9 kcal mol^−1^ nicely agrees with a trend in the matching IC_50_(P*f*3D7) activities of 0.04 µM for **5e** and 0.44 µM for its triazole-based analogue [15], which predicts a difference of ΔΔ*G*_BIND_ = −1.6 kcal mol^−1^. Secondly, computed values evidently distinguish between *O*-substituted and *N*-substituted derivatives, with the latter group involving better binders amid the two families. Lastly, among each set, the calculated Δ*G*_BIND_ values indicate identical binding trends regarding the substituents introduced, which assume *p*-Cl (**5e**) > *p*-F (**5d**) > *p*-H (**5a**), in the same way as the matching *O*-substituted derivatives **6e** > **6d** > **6a**. With this in mind, we can safely conclude that the excellent agreement between computations and experiments lends strong credence to the computational methodology employed.

**Figure 3:**
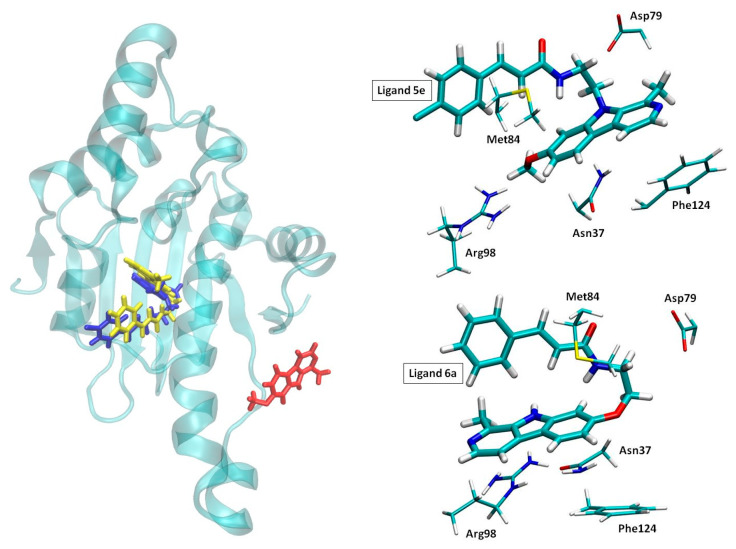
Binding positions of ligands **5e** (in blue) and **6a** (in yellow) within the ATP binding site in *Pf*Hsp90 and harmine (in red) outside of it (left). The interaction of **5e** (top right) and **6a** (bottom right) with the relevant binding site residues.

The calculated Δ*G*_BIND_ value for **5e** is the most exergonic, suggesting it is optimally positioned within *Pf*Hsp90. Its decomposition into contributions from specific residues reveals that **5e** is clearly positioned within the ATP binding site, as its binding is dominated by residues that define this cavity (Figure 3). This holds in particular for Asn37 and Arg98, where the contributions obtained are −2.01 and −1.39 kcal mol^−1^, respectively, closely followed by Phe124, where it is −0.64 kcal mol^−1^. The corresponding MD trajectories show that the tricyclic aromatic ring and the attached –OMe group on **5e** are responsible for these favorable contributions. Specifically, side chains of both Asn37 and Phe124 are located above the aromatic framework of **5e**, forming positive N–H∙∙∙π and π∙∙∙π stacking interactions, respectively, while Arg98 uses its cationic guanidine moiety to donate hydrogen bonding to the ligand’s methoxy oxygen. The overall effect of these three residues is somewhat reduced by the unfavorable effect of Asp79, which is overcome by the contribution of several other residues that promote the binding (Table 2), and which are positioned close to the ATP binding site. The reason behind the negative contribution of Asp79 lies in the repulsion between its carboxylic moiety and the –CH_2_–CH_2_– linker in **5e** connecting the amide substituent with the harmine *N*-position. This might suggest that the parent harmine, containing all of the positively contributing structural elements—both the tricyclic ring and the –OMe group—while being deprived of the mentioned linker, let alone having the acidic N–H instead that could engage in favorable interactions with Asp79, would represent a much better binder than **5e**. However, quite the opposite occurs, as harmine reveals very poor binding properties, displaying a significantly lower Δ*G*_BIND_ (−7.5 kcal mol^−1^) (Table 2), consistent with the significantly higher IC_50_ value of 8.25 μM (Table 1). Moreover, although the relationship between IC_50_ and Δ*G*_BIND_ values is not so straightforward, the IC_50_ value measured for harmine roughly translates to a binding energy of −6.9 kcal mol^−1^, which again confirms the validity of our computational setup and nicely ties into our computational and experimental results. Additionally, the data shown in Table 2 clearly indicate that, aside from a very low ∆*G*_BIND_ value, harmine binds even outside the ATP binding pocket (Figure 3), as none of the residues either defining it or close to it are contributing to the binding. All of these observations underline the importance of the *N-*site of harmine for the inactivation of *Pf*Hsp90, and justify the synthetic strategy employed here, as carefully tailored *N*-substituents can overcome unfavorable interactions with the anionic Asp79 through their positive interactions with the rest of the protein. This valuable insight might provide precious information for further harmine derivatization. Lastly, besides the ATP binding site residues mentioned above, the binding of **5e** is particularly promoted by Met84, which uses its –SMe side chain to form the stabilizing C–H∙∙∙π interactions with the aromatic unit in **5e** (Figure 3), thus allowing for the highest individual contribution among all amino acid residues. 

Interestingly, although *N*-derivatives **5** with different *para*-groups exhibit different antiplasmodial activities (Table 1), we did not observe any significant interactions between moieties placed at this position and the protein (Figure 3). Instead, this site points outside the *Pf*Hsp90. Thus, it is presently unclear what the immediate effect of this position for the ligand affinity is, and how to eventually advance the binding properties with such further substitutions. Still, the extent of this effect is modest and is likely channeled through electron-donating or electron-accepting features of the introduced substituent that modulate the electron density throughout each derivative, which, in turn, affect its interactions with the protein. This conclusion is justified by the data in Table 1, considering that all six *N*-derivatives stretch over only one order of magnitude in the affinities measured, from 0.49 μM in **5a** to 0.04 μM in **5e**, thus confirming only a minor effect of the introduced *para*-substituent. Therefore, it is unlikely that a potential insertion of additional *para*-moieties in **5** will considerably improve the compounds’ activity. Instead, redesigning the employed –CH_2_–CH_2_– linker emerges as a promising strategy that will be exploited in our future work.

Replacing *p*-Cl (**5e**) with *p*-F (**5d**) decreases the IC_50_ value by a factor of two and is supported by the 2.8 kcal mol^−1^ lower binding energy of Δ*G*_BIND_(**5d**) = −38.1 kcal mol^−1^. In **5d**, reduced contributions from the ATP binding site residues are observed, which suggests a slight change in the binding pose, except for Phe124, which benefits from the modified ligand’s positions by gaining a full kcal mol^−1^ relative to **5e**, as a result of the more optimized π∙∙∙π stacking interactions in that case. Lastly, complete removal either of the *para*-halogens, as in **5a**, yields a further reduction in both IC_50_ and Δ*G*_BIND_ values and results in a slightly changed binding pose. The latter appears enough to position the –OMe group away from Arg98, leading to a significant reduction in its binding contribution, while lowering the impact of other residues as well. Given the lowest affinity of **5a** among all *N*-derivatives, these results suggest the predominant role of the introduced *para*-substituent through tuning the position of ligands within the binding site, thus allowing for the optimization of ligand contacts with the protein residues. 

Derivatives **6** have a functionalized harmine –OMe group, while maintaining the unsubstituted secondary amine on the central aromatic ring. In line with the above, this is immediately evident in diminishing the negative contribution of Asp79 from 1.58 kcal mol^−1^ in **5e** to 1.11, 1.30 and even 0.74 kcal mol^−1^ in **6a**, **6d** and **6e**, respectively. Yet, these highly positive values do not indicate any favorable hydrogen bonding among Asp79 carboxylate and the ligands amino group, which was also not observed in our simulations. On the other hand, derivatives **6** are also positioned within the ATP binding site in *Pf*Hsp90, but in a way which does not allow for an efficient interaction with Asp79 (Figure 3). This change in the binding pose is evident in the increase in the contribution of Arg98, which moves from forming hydrogen bonding with the ligand’s methoxy oxygen, as in **5**, into forming cation∙∙∙π interactions with aromatic fragments in the *O*-substituted derivatives **6** that turned out as even more favorable (Table 2). The latter also allows Asn92 to optimize its N–H∙∙∙π interactions with systems **6**, evident in its increased contributions. Other than those, in general, most of the ATP binding site residues and those in their vicinity have reduced contributions relative to the analogous systems **5**, ultimately leading to the lower activities observed for the matching *O*-substituted derivatives **6**. A likely reason for that is the observed tendency of these systems to assume geometries that enable the intramolecular C–H···π stacking interactions among aromatic fragments within the ligands **6** themselves (Figure 3), which diminish a number of potential contacts with the protein. Additionally, data in Table 2 shows a significant drop in the individual contributions of Thr171, Ile173, Leu34 and Val136, which are considerably lower in **6** than in **5**. These residues form a hydrophobic pocket around the unsubstituted –OMe group and vicinal fragments in **5**, thus promoting the binding, which is not possible upon substituting this part in systems **6**, thus explaining the lower affinities of the latter. Overall, this indicates that the *O*-site in the harmine derivatives provides fewer promising opportunities for the synthesis of successful *Pf*Hsp90 binders, in agreement with our experimental data.

## 3. Materials and Methods

### 3.1. Chemistry

#### 3.1.1. General Information

Melting points were determined on a Stuart Melting Point Apparatus (Barloworld Scientific, England, UK) in open capillaries and were uncorrected. FTIR-ATR spectra were recorded using a Fourier-Transform Infrared Attenuated Total Reflection UATR Two Spectrometer (PerkinElmer, Waltham, MA, USA) in the range from 450 to 4000 cm^−1^. ^1^H and ^13^C NMR spectra were recorded on a Bruker Avance III HD operating at 300 or 400 MHz for the ^1^H and 75, 101 or 151 MHz for the ^13^C nuclei (Bruker, Billerica, MA, USA). Samples were measured in DMSO-*d*_6_ solutions at 20 °C in 5 mm NMR tubes. Chemical shifts (*δ*) are reported in parts per million (ppm) using tetramethylsilane (TMS) as a reference in the ^1^H and DMSO residual peak as a reference in the ^13^C spectra (39.51 ppm). Coupling constants (*J*) are reported in hertz (Hz). Mass spectra were recorded on HPLC-MS/MS (HPLC, Agilent Technologies 1200 Series; MS, Agilent Technologies 6410 Triple Quad, San Jose, CA, USA). Mass determination was performed using electrospray ionization (ESI) in a positive mode. Elemental analyses were performed on a CHNS LECO analyzer (LECO Corporation, St. Joseph, MI, USA). Analyses indicated by the symbols of the elements were within ±0.4% of their theoretical values. Microwave-assisted reactions were performed in a microwave reactor CEM Discover (CEM, Matthews, NC, USA) in a glass reaction vessel. All compounds were routinely checked by TLC with silica gel 60F-254 glass plates (Merck KGaA, Darmstadt, Germany) using DCM/methanol 8:1 and 85:15 as the solvent system. Spots were visualized by short-wave UV light (*λ* = 254 nm) and iodine vapor. Column chromatography was performed on silica gel 0.063–0.200 mm (Sigma-Aldrich, St. Louis, MO, USA) with the same eluents used for TLC. All chemicals and solvents were of analytical grade and purchased from commercial sources. CADs were purchased as predominantly *trans* stereoisomers (≥99%). Harmine, acetic acid, hydrochloric acid, cinnamic acid, α-methylcinnamic acid, 2-fluorocinnamic acid, 3-fluorocinnamic acid, 4-fluorocinnamic acid, 4-methoxycinnamic acid and 4-chlorocinnamic acid were purchased from Sigma-Aldrich (St. Louis, MO, USA). Cesium carbonate, 2-(Boc-amino)ethyl bromide, HATU and 3-bromocinnamic acid were purchased from TCI Chemicals (Tokyo, Japan). Hydrobromic acid (47%) was purchased from Merck (Darmstadt, Germany), DIEA from Alfa Aesar (Kandel, Germany), DCM from Fischer Scientific (Waltham, MA, USA), diethyl ether from ITW Reagents (Darmstadt, Germany), anhydrous sodium sulphate from Gram-Mol (Zagreb, Croatia) and *N,N*-dimethylformamide (DMF) from Kemika (Zagreb, Croatia). Harmole was prepared according to the modified literature procedure for harmine using HBr/glacial acetic acid mixture, under MW irradiation [30].

#### 3.1.2. Synthesis of tert-butyl (2-(7-methoxy-1-methyl-9H-pyrido[3,4-b]indol-9-yl)ethyl)carbamate (**1**)

To a stirred solution of harmine (0.200 g, 0.94 mmol) in anhydrous DMF (3 mL) at 75 °C under an argon atmosphere, Cs_2_CO_3_ (1.228 g, 3.77 mmol, 4 equivalents) was added. The resulting suspension was stirred at 75 °C for 20 min, followed by the addition of 2-(Boc-amino)ethyl bromide (0.844 g, 3.77 mmol, 4 equivalents). The reaction mixture was stirred at 75 °C for 24 h, cooled down, poured into H_2_O (40 mL) and extracted with ethyl acetate (3 × 40 mL). The collected organic layers were washed with H_2_O, dried over anhydrous sodium sulfate, filtered, and evaporated under the reduced pressure. After purification by column chromatography (DCM:MeOH = 8:1) and trituration with diethyl ether, 0.195 g (58%) of **1** was obtained; mp 197.5–199 °C; IR (ATR, *ν*/cm^−1^) 3183, 3065, 3013, 2993, 2969, 2937, 1701, 1623, 1567, 1502, 1447, 1409, 1366, 1343, 1330, 1313, 1278, 1247, 1195, 1166, 1146, 1124, 1092, 1048, 1018, 970, 944, 876, 841, 803, 768, 727, 684, 639, 598, 559, 534; ^1^H NMR (DMSO-*d*_6_) *δ* 8.15 (d, 1H, *J* = 5.1 Hz), 8.07 (d, 1H, *J* = 8.5 Hz), 7.85 (d, 1H, *J* = 5.0 Hz), 7.22 (s, 1H), 7.02 (t, 1H, *J* = 5.2 Hz), 6.87 (dd, 1H, *J* = 8.4, 1.6 Hz), 4.55 (t, 2H, *J* = 6.6 Hz), 3.92 (s, 3H), 3.33–3.31 (m, 2H), 2.95 (s, 3H), 1.29 (s, 8H), 1.01 (s, 1H); ^13^C NMR (DMSO-*d*_6_) *δ* 160.45, 155.62, 142.84, 140.46, 137.63, 134.70, 128.39, 122.20, 114.35, 112.10, 108.95, 93.80, 77.77, 55.47, 43.98, 39.97, 28.01, 22.99; ESI-MS: *m/z* 356.2 (M + 1)^+^.

#### 3.1.3. Synthesis of tert-butyl (2-((1-methyl-9H-pyrido[3,4-b]indol-7-yl)oxy)ethyl)carbamate (**2**)

To a stirred solution of harmole (0.257 g, 1.30 mmol) in anhydrous DMF (3 mL), under an argon atmosphere, Cs_2_CO_3_ (0.570 g, 1.75 mmol, 1.35 equivalents) was added. The resulting suspension was stirred at room temperature for 20 min, followed by the addition of 2-(Boc-amino)ethyl bromide (0.755 g, 3.37 mmol, 2.6 equivalents). The reaction mixture was stirred at 110 °C for 4 h, cooled down, poured into H_2_O (30 mL) and extracted with ethyl acetate (3 × 40 mL). The collected organic layers were washed with H_2_O, dried over anhydrous sodium sulfate, filtered, and evaporated under the reduced pressure. After purification by column chromatography (DCM:MeOH = 8:1) and trituration with diethyl ether, 0.278 g (63%) of **2** was obtained; mp 189.5–190.5 °C; IR (ATR, *ν*/cm^−1^) 3380, 3134, 3052, 2978, 2944, 2872, 2764, 2726, 2582, 2406, 1692, 1634, 1568, 1530, 1480, 1448, 1394, 1368, 1334, 1296, 1260, 1174, 1114, 1052, 1010, 964, 872, 846, 820, 788, 720, 686, 634, 604, 522; ^1^H NMR (DMSO-*d*_6_) *δ* 11.42 (s, 1H), 8.15 (d, 1H, *J* = 5.3 Hz), 8.05 (d, 1H, *J* = 8.6 Hz), 7.81 (d, 1H, *J* = 5.3 Hz), 7.06 (t, 1H, *J* = 5.4 Hz), 7.00 (d, 1H, *J* = 2.1 Hz), 6.85 (dd, 1H, *J* = 8.7, 2.2 Hz), 4.08 (t, 2H, *J* = 5.8 Hz), 3.41–3.35 (m, 2H), 2.73 (s, 3H), 1.40 (s, 9H); ^13^C NMR (DMSO-*d*_6_) *δ* 159.21, 155.72, 141.91, 141.20, 137.58, 134.54, 127.25, 122.65, 114.95, 111.95, 109.32, 95.38, 77.79, 66.65, 28.22, 20.25; ESI-MS: *m/z* 342.3 (M + 1)^+^.

#### 3.1.4. General Procedure for the Synthesis of Amines **3** and **4**

A solution of the corresponding compound **1** or **2** (0.70 mmol) and 1.76 mL 4 M HCl (7 mmol) in MeOH (4 mL) was stirred at 50 °C for 16 h (compound **1**) or 2 h (compound **2**). Upon completion, solvent was removed under the reduced pressure. The residue was dissolved in H_2_O (20 mL), basified to pH 12 with 5% NaOH, and extracted with ethyl acetate (5 × 40 mL). The collected organic layers were dried over anhydrous sodium sulfate, filtered, and evaporated under reduced pressure. The crude product was triturated with diethyl ether.

##### 2-(7-Methoxy-1-methyl-9*H*-pyrido[3,4-*b*]indol-9-yl)ethan-1-amine (**3**)

Yield: 0.179 g, (76%); mp 133.5–135.5 °C; IR (ATR, *ν*/cm^−1^); 3354, 3327, 3274, 3054, 3039, 2966, 2932, 2837, 1751, 1621, 1563, 1496, 1443, 1404, 1343, 1302, 1283, 1236, 1219, 1151, 1136, 1117, 1089, 1040, 1021, 973, 941, 922, 887, 848, 815, 768, 723, 643, 599, 551, 528; ^1^H NMR (DMSO-*d*_6_) *δ* 8.16 (d, 1H, *J* = 5.2 Hz), 8.07 (d, 1H, *J* = 8.6 Hz), 7.86 (d, 1H, *J* = 5.2 Hz), 7.24 (d, 1H, *J* = 2.1 Hz), 6.87 (dd, 1H, *J* = 8.6, 2.2 Hz), 4.54 (t, 2H, *J* = 7.3 Hz), 3.91 (s, 3H), 2.97 (s, 3H), 2.93 (t, 2H, 2’, *J* = 7.3 Hz); ^13^C NMR (DMSO-*d*_6_) *δ* 160.51, 142.95, 140.67, 137.64, 134.79, 128.22, 122.33, 114.17, 112.23, 109.14, 93.79, 55.63, 47.15, 42.11, 23.33; ESI-MS: *m/z* 256.3 (M + 1)^+^.

##### 2-((1-Methyl-9*H*-pyrido[3,4-*b*]indol-7-yl)oxy)ethan-1-amine (**4**)

Yield: 0.169 g, (66%); mp 172.5–173 °C; IR (ATR, ν/cm^−1^) 3954, 3908, 3838, 3786, 3716, 3656, 3530, 3394, 3332, 3250, 3156, 3068, 2930, 2864,2774, 2372, 1892, 1756, 1720, 1628, 1568, 1486, 1444, 1326, 1284, 1238, 1180, 1104, 1028, 996, 908, 860, 812, 636, 590; ^1^H NMR (DMSO-*d*_6_) *δ* 11.38 (s, 1H), 8.15 (d, 1H, *J* = 5.3 Hz), 8.04 (d, 1H, *J* = 8.6 Hz), 7.79 (d, 1H, *J* = 5.3 Hz), 7.01 (d, 1H, *J* = 2.2 Hz), 6.85 (dd, 1H, *J* = 8.6, 2.2 Hz), 4.03 (t, 2H, *J* = 5.7 Hz), 2.95 (t, 2H, *J* = 5.7 Hz), 2.73 (s, 3H), 1.57 (s, 2H); ^13^C NMR (DMSO-*d*_6_) *δ* 159.47, 141.90, 141.23, 137.73, 134.53, 127.19, 122.57, 114.82, 111.89, 109.37, 95.27, 70.46, 41.02, 20.32; ESI-MS: *m/z* 242.2 (M + 1)^+^.

#### 3.1.5. General Procedure for the Synthesis of Harmicines **5a**–**f**

A solution of CAD (0.18 mmol), DIEA (0.061 mL, 0.35 mmol) and HATU (0.067 g, 0.18 mmol) in DCM (3 mL) was stirred at room temperature for 15 min, followed by the addition of amine **3** (0.045 g, 0.18 mmol). The resulting solution was stirred at room temperature for 2 h. Purification was performed by either Method A or Method B.

Method A: The reaction mixture was extracted with brine (2 × 20 mL) and water (1 × 20 mL). The organic layer was dried over anhydrous sodium sulfate, filtered and evaporated under the reduced pressure. The crude product was purified by column chromatography (DCM:MeOH = 8:1) and triturated with diethyl ether.

Method B: The resulting precipitate was filtered off. The mother liquor was extracted with brine (2 × 20 mL) and water (1 × 20 mL). The organic layer was dried over anhydrous sodium sulfate, filtered, evaporated under the reduced pressure and residue combined with the filtered precipitate. The crude product was purified by column chromatography (DCM:MeOH = 8:1) and triturated with diethyl ether.

##### *N*-(2-(7-methoxy-1-methyl-9*H*-pyrido[3,4-*b*]indol-9-yl)ethyl)cinnamamide (**5a**)

CAD: 0.026 g of *trans-*cinnamic acid; purification: Method A; yield: 0.034 g, (51%); mp 201.5–203 °C; IR (ATR, *ν*/cm^−1^) 3202, 3027, 2968, 2926, 2852, 1672, 1622, 1566, 1532, 1496, 1447, 1405, 1340, 1306, 1282, 1247, 1218, 1196, 1175, 1138, 1091, 1043, 1020, 972, 938, 856, 814, 762, 679, 641, 597, 555, 485; ^1^H NMR (DMSO-*d*_6_) *δ* 8.41 (t, 1H, *J* = 5.9 Hz), 8.18 (d, 1H, *J* = 5.2 Hz), 8.09 (d, 1H, *J* = 8.6 Hz), 7.88 (d, 1H, *J* = 5.2 Hz), 7.57–7.55 (m, 2H), 7.48–7.36 (m, 4H), 7.27 (d, 1H, *J* = 1.9 Hz), 6.87 (dd, 1H, *J* = 8.6, 2.0 Hz), 6.54 (d, 1H, *J* = 15.8 Hz), 4.67 (t, 2H, *J* = 6.8 Hz), 3.90 (s, 3H), 3.59 (q, 2H, *J* = 6.5 Hz), 2.99 (s, 3H); ^13^C NMR (DMSO-*d*_6_) *δ* 165.72, 160.49, 142.90, 140.64, 139.16, 137.85, 134.74, 134.64, 129.56, 128.94, 128.46, 127.57, 122.37, 121.65, 114.27, 112.25, 109.40, 93.49, 55.38, 43.36, 39.10, 23.09; ESI-MS: *m/z* 386.4 (M + 1)^+^; Anal. Calcd. for C_24_H_23_N_3_O_2_: C, 74.78; H, 6.01; N, 10.90, found: C, 74.88; H, 6.21; N, 10.75.

##### (*E*)-3-(3-fluorophenyl)-*N*-(2-(7-methoxy-1-methyl-9*H*-pyrido[3,4-*b*]indol-9-yl)ethyl)acrylamide (**5b**)

CAD: 0.029 g of 3-fluorocinnamic acid; purification: Method A; yield: 0.033 g, (47%); mp 226.5–228 °C; IR (ATR, *ν*/cm^−1^); 3661, 3599, 3182, 3009, 2931, 2838, 1671, 1623, 1584, 1538, 1505, 1445, 1412, 1343, 1295, 1240, 1182, 1141, 1095, 1043, 1017, 976, 848, 808, 783, 729, 668, 556, 517; ^1^H NMR (DMSO-*d*_6_) *δ* 8.42 (t, 1H, *J* = 5.9 Hz), 8.18 (d, 1H, *J* = 5.2 Hz), 8.09 (d, 1H, *J* = 8.6 Hz), 7.89 (d, 1H, *J* = 5.2 Hz), 7.49–7.39 (m, 4H), 7.27 (d, 1H, *J* = 2.0 Hz), 7.25–7.18 (m, 1H), 6.87 (dd, 1H, *J* = 8.6, 2.1 Hz), 6.59 (d, 1H, *J* = 15.8 Hz), 4.67 (t, 2H, *J* = 6.9 Hz), 3.90 (s, 3H), 3.59 (q, 2H, *J* = 6.5 Hz), 2.99 (s, 3H); ^13^C NMR (DMSO-*d*_6_) *δ* 165.44, 162.45 (d, *J* = 243.8 Hz), 160.51, 142.91, 140.62, 137.89, 137.80, 137.35 (d, *J* = 7.9 Hz), 134.63, 130.90 (d, *J* = 8.4 Hz), 128.50, 123.76, 123.20, 122.40, 116.23 (d, *J* = 21.3 Hz), 114.28, 113.97 (d, *J* = 21.8 Hz), 112.27, 109.40, 93.51, 55.39, 43.33, 39.10, 23.05; ESI-MS: *m/z* 404.4 (M + 1)^+^; Anal. Calcd. for C_24_H_22_FN_3_O_2_: C, 71.45; H, 5.50; N, 10.42, found: C, 71.26; H, 5.79; N, 10.59.

##### (*E*)-3-(3-bromophenyl)-*N*-(2-(7-methoxy-1-methyl-9*H*-pyrido[3,4-*b*]indol-9-yl)ethyl)acrylamide (**5c**)

CAD: 0.040 g of 3-bromocinnamic acid; purification: Method B; yield: 0.057 g, (70%); mp 225–226 °C; IR (ATR, *ν*/cm^−1^); 3180, 3051, 3021, 2975, 2932, 2901, 2864, 2792, 2559, 1944, 1875, 1679, 1621, 1565, 1498, 1468, 1450, 1410, 1377, 1345, 1305, 1251, 1223, 1195, 1138, 1106, 1043, 1018, 980, 941, 911, 885, 862, 812, 786, 729, 671, 648, 614, 599, 555, 537, 515; ^1^H NMR (DMSO-*d*_6_) *δ* 8.39 (t, 1H, *J* = 6.0 Hz), 8.18 (d, 1H, *J* = 5.2 Hz), 8.09 (d, 1H, *J* = 8.6 Hz), 7.89 (d, 1H, *J* = 5.2 Hz), 7.77 (t, 1H, *J* = 1.7 Hz), 7.57 (dd, 2H, *J* = 8.1, 1.4 Hz), 7.44–7.35 (m, 2H), 7.26 (d, 1H, *J* = 2.1 Hz), 6.87 (dd, 1H, *J* = 8.6, 2.2 Hz), 6.59 (d, 1H, *J* = 15.8 Hz), 4.67 (t, 2H, *J* = 6.9 Hz), 3.90 (s, 3H), 3.60 (q, 2H, *J* = 6.5 Hz), 2.99 (s, 3H); ^13^C NMR (DMSO-*d*_6_) 165.39, 160.52, 142.92, 140.62, 137.80, 137.57, 137.33, 134.64, 132.09, 131.04, 130.13, 128.52, 126.43, 123.32, 122.41, 122.27, 114.29, 112.29, 109.39, 93.54, 55.41, 43.34, 39.10, 23.05; ESI-MS: *m/z*, 464.3 (M + 1)^+^, 466.3 (M + 1)^+^; Anal. Calcd. for C_24_H_22_BrN_3_O_2_: C, 62.08; H, 4.78; N, 9.05, found: C, 62.31; H, 4.89; N, 9.27.

##### (*E*)-3-(4-fluorophenyl)-*N*-(2-(7-methoxy-1-methyl-9*H*-pyrido[3,4-*b*]indol-9-yl)ethyl)acrylamide (**5d**)

CAD: 0.029 g of 4-fluorocinnamic acid; purification: Method B; yield: 0.042 g, (57%); mp 202.5–204 °C; IR (ATR, *ν*/cm^−1^); 3664, 3583, 3438, 3207, 3083, 2985, 2938, 2756, 2723, 1656, 1622, 1601, 1545, 1508, 1471, 1449, 1416, 1390, 1370, 1345, 1292, 1256, 1223, 1203, 1181, 1163, 1141, 1098, 1045, 1018, 976, 944, 836, 741, 722, 644, 557, 530, 511; ^1^H NMR (DMSO-*d*_6_) *δ* 8.40 (s, 1H), 8.19 (d, 1H, *J* = 4.3 Hz), 8.11 (d, 1H, *J* = 8.4 Hz), 7.92 (d, 1H, *J* = 4.2 Hz), 7.62 (t, 2H, *J* = 6.6 Hz), 7.45 (d, 1H, *J* = 15.7 Hz), 7.28–7.23 (m, 3H), 6.88 (d, 1H, *J* = 8.0 Hz), 6.49 (d, 1H, *J* = 15.7 Hz), 4.67 (t, 2H, *J* = 6.7 Hz), 3.90 (s, 3H), 3.59 (q, 2H, *J* = 6.8 Hz), 3.00 (s, 3H); ^13^C NMR (DMSO-*d*_6_) *δ* 165.69, 162.76 (d, *J* = 247.0 Hz), 160.67, 143.12, 140.44, 138.01, 137.34, 134.59, 131.38 (d, *J* = 2.5 Hz), 129.77 (d, *J* = 8.3 Hz), 128.78, 122.52, 121.53, 115.93 (d, *J* = 21.7 Hz), 114.22, 112.40, 109.62, 93.52, 55.44, 43.40, 39.09, 22.76; ESI-MS: *m/z* 404.2 (M + 1)^+^; Anal. Calcd. for C_24_H_22_FN_3_O_2_: C, 71.45; H, 5.50; N, 10.42, found: C, 71.58; H, 5.37; N, 10.48.

##### (*E*)-3-(4-chlorophenyl)-*N*-(2-(7-methoxy-1-methyl-9*H*-pyrido[3,4-*b*]indol-9-yl)ethyl)acrylamide (**5e**)

CAD: 0.032 g of 4-chlorocinnamic acid; purification: Method B; yield: 0.039 g, (53%); mp 245 °C (decomp.); IR (ATR, *ν*/cm^−1^) 3195, 3103, 3025, 2998, 2928, 2835, 1672, 1621, 1558, 1493, 1442, 1407, 1338, 1284, 1254, 1200, 1138, 1086, 1045, 977, 947, 799, 748, 708, 680, 634, 593, 550, 497; ^1^H NMR (DMSO-*d*_6_) *δ* 8.41 (t, 1H, *J* = 5.9 Hz), 8.17 (d, 1H, *J* = 5.2 Hz), 8.09 (d, 1H, *J* = 8.6 Hz), 7.88 (d, 1H, *J* = 5.2 Hz), 7.59 (d, 2H*, J* = 8.5 Hz), 7.49–7.42 (m, 3H), 7.26 (d, 1H, *J* = 2.0 Hz), 6.87 (dd, 1H, *J* = 8.6, 2.1 Hz), 6.55 (d, 1H, *J* = 15.8 Hz), 4.67 (t, 2H, *J* = 6.8 Hz), 3.89 (s, 3H), 3.59 (q, 2H, *J* = 6.5 Hz), 2.99 (s, 3H); ^13^C NMR (DMSO-*d*_6_) *δ* 165.53, 160.50, 142.90, 140.63, 137.83, 134.64, 133.99, 133.70, 129.29, 128.98, 128.48, 122.44, 122.38, 114.28, 112.26, 109.39, 93.50, 55.39, 43.34, 39.10, 23.07; ESI-MS: *m/z* 420.3 (M + 1)^+^; Anal. Calcd. for C_24_H_22_ClN_3_O_2_: C, 68.65; H, 5.28; N, 10.01, found: C, 68.90; H, 5.11; N, 10.23.

##### (*E*)-*N*-(2-(7-methoxy-1-methyl-9*H*-pyrido[3,4-*b*]indol-9-yl)ethyl)-3-(4-methoxyphenyl)acrylamide (**5f**)

CAD: 0.031 g of 4-methoxycinnamic acid; purification: Method A; yield: 0.046 g, (61%); mp 216–217.5 °C; IR (ATR, *ν*/cm^−1^); 3212, 3102, 3040, 2993, 2973, 2940, 2919, 2840, 1667, 1620, 1604, 1558, 1514, 1474, 1443, 1409, 1382, 1338, 1306, 1283, 1254, 1229, 1198, 1173, 1138, 1096, 1033, 979, 949, 827, 801, 780, 731, 701, 684, 635, 592, 541, 521; ^1^H NMR (DMSO-*d*_6_) *δ* 8.31 (t, 1H, *J* = 5.9 Hz), 8.17 (d, 1H, *J* = 5.2 Hz), 8.09 (d, 1H, *J* = 8.6 Hz), 7.88 (d, 1H, *J* = 5.2 Hz), 7.51 (d, 2H, *J* = 8.8 Hz), 7.41 (d, 1H, *J* = 15.8 Hz), 7.27 (d, 1H, *J* = 2.1 Hz), 6.97 (d, 2H, *J* = 8.8 Hz), 6.87 (dd, 1H, *J* = 8.6, 2.2 Hz), 6.40 (d, 1H, *J* = 15.8 Hz), 4.66 (t, 2H, *J* = 6.9 Hz), 3.90 (s, 3H), 3.79 (s, 3H), 3.58 (q, 2H, *J* = 6.6 Hz), 2.99 (s, 3H); ^13^C NMR (DMSO-*d*_6_) *δ* 166.04, 160.51, 160.41, 142.92, 140.63, 138.88, 137.81, 134.64, 129.17, 128.47, 127.30, 122.38, 119.15, 114.39, 114.26, 112.26, 109.43, 93.49, 55.38, 55.26, 43.42, 39.09, 23.06; ESI-MS: *m/z* 416.3 (M + 1)^+^; Anal. Calcd. for C_25_H_25_N_3_O_3_: C, 72.27; H, 6.07; N, 10.11, found: C, 72.43; H, 6.32; N, 10.35.

#### 3.1.6. General Procedure for the Synthesis of Harmicines **6a–h**

A solution of CAD (0.20 mmol), DIEA (0.070 mL, 0.40 mmol) and HATU (0.077 g, 0.20 mmol) in DCM (4 mL) was stirred at room temperature for 15 min, followed by the addition of amine **4** (0.049 g, 0.20 mmol). The resulting solution was stirred at room temperature for 1 h. The formed precipitate was filtered off. After purification by column chromatography (DCM:MeOH = 85:15) and trituration with diethyl ether, compounds **6a**–**h** were obtained.

##### *N*-(2-((1-methyl-9*H*-pyrido[3,4-*b*]indol-7-yl)oxy)ethyl)cinnamamide (**6a**)

CAD: 0.030 g of *trans-*cinnamic acid; yield: 0.045 g, (60%); mp 151.5 °C (decomp.); IR (ATR, *ν*/cm^−1^) 3994, 3908, 3838, 3784, 3718, 3656, 3424, 3222, 3060, 2972, 2932, 2874, 2768, 2372, 1898, 1720, 1630, 1574, 1544, 1486, 1450, 1392, 1332, 1280, 1234, 1174, 1110, 1056, 976, 812, 764, 684, 596; ^1^H NMR (DMSO-*d*_6_) *δ* 11.66 (s, 1H), 8.47 (t, 1H, *J* = 5.3 Hz,), 8.19 (d, 1H, *J* = 5.4 Hz), 8.11 (d, 1H, *J* = 8.7 Hz), 7.90 (d, 1H, *J* = 5.4 Hz), 7.57 (d, 2H, *J* = 7.0 Hz), 7.48 (d, 1H, *J* = 15.8 Hz), 7.44–7.35 (m, 3H), 7.07 (d, 1H, *J* = 1.8 Hz), 6.91 (dd, 1H, *J* = 8.7, 2.0 Hz), 6.73 (d, 1H, *J* = 15.8 Hz), 4.20 (t, 2H, *J* = 5.4 Hz), 3.65 (q, 2H, 5.3 Hz), 2.77 (s, 3H); ^13^C NMR (DMSO-*d*_6_) *δ* 165.29, 159.52, 142.38, 140.70, 138.87, 136.37, 134.87, 134.45, 129.46, 128.92, 127.89, 127.52, 122.95, 122.02, 114.84, 112.25, 109.80, 95.39, 66.68, 38.51, 19.66; ESI-MS: *m/z* 372.2 (M + 1)^+^; Anal. Calcd. for C_23_H_21_N_3_O_2_: C, 74.37; H, 5.70; N, 11.31, found: C, 74.67; H, 5.75; N, 11.22.

##### (*E*)-3-(3-fluorophenyl)-*N*-(2-((1-methyl-9*H*-pyrido[3,4-*b*]indol-7-yl)oxy)ethyl)acrylamide (**6b**)

CAD: 0.034 g of 3-fluorocinnamic acid; yield: 0.053 g, (67%); mp 210–212.5 °C; (ATR, *ν*/cm^−1^); 3954, 3908, 3838, 3784, 3718, 3656, 3256, 3070, 2940, 2866, 2812, 2370, 1872, 1778, 1720, 1670, 1630, 1580, 1486, 1444, 1344, 1250, 1178, 1110, 1060, 1028, 964, 852, 784, 670, 624, 594, 528; ^1^H NMR (DMSO-*d*_6_) *δ* 11.47 (s, 1H), 8.47 (t, 1H, *J* = 5.5 Hz), 8.16 (d, 1H, *J* = 5.3 Hz), 8.08 (d, 1H, *J* = 8.7 Hz), 7.83 (d, 1H, *J* = 5.3 Hz), 7.50–7.40 (m, 4H), 7.21 (ddd, 1H, *J* = 9.9, 3.9, 2.0 Hz), 7.05 (d, 1H, *J* = 2.1 Hz), 6.89 (dd, 1H, *J* = 8.7, 2.2 Hz), 6.77 (d, 1H, *J* = 15.8 Hz), 4.19 (t, 2H, *J* = 5.4 Hz), 3.65 (q, 2H, *J* = 5.4 Hz), 2.74 (s, 3H); ^13^C NMR (DMSO-*d*_6_) *δ* 165.00, 162.44 (d, *J* = 243.8 Hz), 159.22, 141.97, 141.16, 137.59, 137.47, 137.42, 134.54, 130.88 (d, *J* = 8.4 Hz), 127.33, 123.65, 123.60, 122.72, 116.12 (d, *J* = 21.3 Hz), 115.00, 113.92 (d, *J* = 21.8 Hz), 112.01, 109.42, 95.42, 66.62, 38.58, 20.17; ESI-MS: *m/z* 390.2 (M + 1)^+^; Anal. Calcd. for C_23_H_20_FN_3_O_2_: C, 70.94; H, 5.18; N, 10.79, found: C, 70.83; H, 5.27; N, 10.99.

##### (*E*)-3-(3-bromophenyl)-*N*-(2-((1-methyl-9*H*-pyrido[3,4-*b*]indol-7-yl)oxy)ethyl)acrylamide (**6c**)

CAD: 0.046 g of 3-bromocinnamic acid; yield: 0.043 g, (47%); mp 135–137.5 °C; IR (ATR, *ν*/cm^−1^) 3908, 3840, 3784, 3718, 3656, 3424, 3206, 3056, 2970, 2932, 2872, 2774, 2374, 1874, 1720, 1666, 1630, 1572, 1450, 1338, 1282, 1234, 1168, 1110, 1054, 972, 846, 786, 668, 602; ^1^H NMR (DMSO-*d*_6_) *δ* 11.48 (s, 1H), 8.44 (t, 1H, *J* = 5.5 Hz), 8.16 (d, 1H, *J* = 5.3 Hz), 8.08 (d, 1H, *J* = 8.7 Hz), 7.83 (d, 1H, *J* = 5.3 Hz), 7.79 (t, 1H, *J* = 1.5 Hz), 7.60–7.55 (m, 2H), 7.45 (d, 1H, *J* = 15.8 Hz), 7.38 (t, 1H, *J* = 7.9 Hz), 7.05 (d, 1H, *J* = 2.1 Hz), 6.89 (dd, 1H, *J* = 8.7, 2.2 Hz), 6.78 (d, 1H, *J* = 15.8 Hz), 4.19 (t, 2H, *J* = 5.4 Hz), 3.65 (q, 2H, *J* = 5.4 Hz), 2.74 (s, 3H); ^13^C NMR (DMSO-*d*_6_) *δ* 164.93, 159.21, 141.97, 141.14, 137.47, 137.39, 137.22, 134.53, 131.97, 131.01, 130.04, 127.34, 126.40, 123.72, 122.72, 122.24, 114.99, 112.01, 109.42, 95.42, 66.62, 38.57, 20.16; ESI-MS: *m/z* 450.1 (M + 1)^+^, 452.1 (M + 1)^+^; Anal. Calcd. for C_23_H_20_BrN_3_O_22_: C, 61.34; H, 4.48; N, 9.33, C, 61.29; H, 4.67; N, 9.68.

##### (*E*)-3-(4-fluorophenyl)-*N*-(2-((1-methyl-9*H*-pyrido[3,4-*b*]indol-7-yl)oxy)ethyl)acrylamide (**6d**)

CAD: 0.034 g of 4-fluorocinnamic acid; yield: 0.057 g, (73%); mp 199.5–200 °C; IR (ATR, *ν*/cm^−1^) 3952, 3908, 3840, 3786, 3718, 3634, 3572, 3430, 3248, 3072, 2940, 2866, 2812, 2600, 2366, 2050, 1872, 1720, 1668, 1630, 1576, 1508, 1456, 1344, 1278, 1230, 1178, 1110, 1030, 694, 796, 674, 626, 594, 506; ^1^H NMR (DMSO-*d*_6_) *δ* 11.47 (s, 1H), 8.44 (t, 1H, *J* = 5.5 Hz), 8.16 (d, 1H, *J* = 5.3 Hz), 8.08 (d, 1H, *J* = 8.7 Hz), 7.83 (d, 1H, *J* = 5.3 Hz), 7.66–7.60 (m, 2H), 7.48 (d, 1H, *J* = 15.8 Hz), 7.29–7.22 (m, 2H), 7.05 (d, 1H, *J* = 2.1 Hz), 6.89 (dd, 1H, *J* = 8.7, 2.2 Hz), 6.68 (d, 1H, *J* = 15.8 Hz), 4.19 (t, 2H, *J* = 5.5 Hz), 3.65 (q, 2H, *J* = 5.4 Hz), 2.74 (s, 3H); ^13^C NMR (DMSO-*d*_6_) *δ* 165.23, 162.69 (d, *J* = 247.1 Hz), 159.23, 141.98, 141.16, 137.69, 137.43, 134.54, 131.51 (d, *J* = 2.7 Hz), 129.68 (d, *J* = 8.4 Hz), 127.34, 122.73, 121.92, 115.90 (d, *J* = 21.7 Hz), 114.99, 112.01, 109.43, 95.42, 66.66, 38.55, 20.18; ESI-MS: *m/z* 390.2 (M + 1)^+^; Anal. Calcd. for C_23_H_20_FN_3_O_2_: C, 70.94; H, 5.18; N, 10.79, found: C, 70.81; H, 5.39; N, 10.63.

##### (*E*)-3-(4-chlorophenyl)-*N*-(2-((1-methyl-9*H*-pyrido[3,4-*b*]indol-7-yl)oxy)ethyl)acrylamide (**6e**)

CAD: 0.037 g of 4-chlorocinnamic acid; yield: 0.052 g, (64%); mp 230.5–234 °C; IR (ATR, *ν*/cm^−1^) 3908, 3838, 3784, 3716, 3656, 3268, 3044, 2942, 2880, 2774, 2372, 1720, 1662, 1626, 1560, 1488, 1450, 1330, 1284, 1234, 1168, 1102, 982, 874, 820, 740, 654, 588; ^1^H NMR (DMSO-*d*_6_) *δ* 11.68 (s, 1H), 8.49 (t, 1H, *J* = 5.5 Hz), 8.19 (d, 1H, *J* = 5.5 Hz), 8.11 (d, 1H, *J* = 8.7 Hz), 7.91 (d, 1H, *J* = 5.4 Hz), 7.60 (d, 2H, *J* = 8.5 Hz), 7.49–7.45 (m, 3H), 7.07 (d, 1H, *J* = 2.1 Hz), 6.91 (dd, 1H, *J* = 8.7, 2.2 Hz), 6.74 (d, 1H, *J* = 15.8 Hz), 4.20 (t, 2H, *J* = 5.4 Hz), 3.65 (q, 2H, *J* = 5.4 Hz), 2.77 (s, 3H); ^13^C NMR (DMSO-*d*_6_) *δ* 165.09, 159.53, 142.40, 140.66, 137.51, 136.29, 134.44, 133.87, 133.84, 129.22, 128.95, 127.92, 122.96, 122.83, 114.84, 112.26, 109.81, 95.38, 66.66, 38.53, 19.63; ESI-MS: *m/z* 390.2 (M + 1)^+^; Anal. Calcd. for C_23_H_20_ClN_3_O_2_: C, 68.06; H, 4.97; N, 10.35, found: C, 68.18; H, 4.84; N, 10.44.

##### (*E*)-3-(4-methoxyphenyl)-*N*-(2-((1-methyl-9*H*-pyrido[3,4-*b*]indol-7-yl)oxy)ethyl)acrylamide (**6f**)

CAD: 0.036 g of 4-methoxycinnamic acid; yield: 0.051 g, (63%); mp 191–194.5 °C; (ATR, *ν*/cm^−1^); 3976, 3908, 3838, 3784, 3718, 3656, 3432, 3258, 3070, 2938, 2770, 2368, 2050, 1874, 1720, 1602, 1546, 1514, 1448, 1280, 1174, 1108, 1030, 984, 872, 828, 744, 714, 674, 588, 554, 518; ^1^H NMR (DMSO-*d*_6_) *δ* 11.46 (s, 1H), 8.35 (t, 1H, *J* = 5.5 Hz), 8.16 (d, 1H, *J* = 5.3 Hz), 8.07 (d, 1H, *J* = 8.7 Hz), 7.83 (d, 1H, *J* = 5.3 Hz), 7.52 (d, 2H, *J* = 8.8 Hz), 7.43 (d, 1H, *J* = 15.8 Hz), 7.05 (d, 1H, *J* = 2.1 Hz), 6.98 (d, 2H, *J* = 8.8 Hz), 6.89 (dd, 1H, *J* = 8.7, 2.2 Hz), 6.58 (d, 1H, *J* = 15.8 Hz), 4.18 (t, 2H, *J* = 5.5 Hz), 3.79 (s, 3H), 3.64 (q, 2H, *J* = 5.4 Hz), 2.73 (s, 3H); ^13^C NMR (DMSO-*d*_6_) *δ* 165.60, 160.33, 159.23, 141.96, 141.18, 138.58, 137.48, 134.54, 129.10, 127.43, 127.31, 122.71, 119.52, 114.99, 114.38, 112.00, 109.41, 95.41, 66.70, 55.24, 38.50, 20.21; ESI-MS: *m/z* 402.2 (M + 1)^+^; Anal. Calcd. for C_24_H_23_N_3_O_3_: C, 71.80; H, 5.77; N, 10.47, found: C, C, 71.89; H, 5.54; N, 10.36.

##### (*E*)-3-(2-fluorophenyl)-*N*-(2-((1-methyl-9*H*-pyrido[3,4-*b*]indol-7-yl)oxy)ethyl)acrylamide (**6g**)

CAD: 0.034 g of 2-fluorocinnamic acid; yield: 0.061 g, (77%); mp 241.5 °C (decomp.); IR (ATR, *ν*/cm^−1^) 3838, 3784, 3716, 3636, 3570, 3252, 3066, 2942, 2864, 2818, 2600, 1866, 1668, 1630, 1576, 1486, 1456, 1344, 1278, 1234, 1180, 1100, 1060, 1022, 964, 850, 812, 750, 676, 626, 594; ^1^H NMR (DMSO-*d*_6_) *δ* 11.47 (s, 1H), 8.60 (t, 1H, *J* = 5.4 Hz), 8.15 (d, 1H, *J* = 5.3 Hz), 8.07 (d, 1H, *J* = 8.6 Hz), 7.81 (d, 1H, *J* = 5.3 Hz), 7.70–7.63 (m, 1H, 7.55 (d, 1H, *J* = 16.0 Hz), 7.48–7.39 (m, 1H), 7.28 (dd, 2H, *J* = 15.7, 8.3 Hz), 7.06 (d, 1H, *J* = 2.0 Hz), 6.88 (dd, 1H, *J* = 8.7, 2.1 Hz), 6.84 (d, 1H, *J* = 16.0 Hz), 4.19 (t, 2H, *J* = 5.4 Hz), 3.65 (q, 2H, *J* = 5.4 Hz), 2.73 (s, 3H); ^13^C NMR (DMSO-*d*_6_) *δ* 165.05, 160.45 (d, *J* = 250.4 Hz), 159.12, 141.87, 141.31, 137.72, 134.57, 131.35, 131.23, 129.08, 127.15, 124.97, 124.85 (d, *J* = 5.9 Hz), 122.65, 122.49 (d, *J* = 11.5 Hz), 116.08 (d, *J* = 21.8 Hz), 115.03, 111.93, 109.30, 95.44, 66.57, 38.61, 20.35; ESI-MS: m/z 390.3 (M + 1)^+^; Anal. Calcd. for C_23_H_20_FN_3_O_2_: C, 70.94; H, 5.18; N, 10.79, found: C, 70.99; H, 4.97; N, 11.01.

##### (*E*)-2-methyl-*N*-(2-((1-methyl-9*H*-pyrido[3,4-*b*]indol-7-yl)oxy)ethyl)-3-phenylacrylamide (**6h**)

CAD: 0.033 g of α-methylcinnamic acid; yield: 0.047 g, (61%); mp 221.5–222.5 °C; (ATR, *ν*/cm^−1^) 3996, 3954, 3908, 3838, 3786, 3716, 3656, 3432, 3202, 3024, 2940, 2880, 2800, 2596, 2512, 2370, 1952, 1884, 1776, 1720, 1630, 1538, 1484, 1446, 1276, 1236, 1174, 1106, 1070, 972, 926, 850, 814, 760, 698, 664, 588, 516; ^1^H NMR (DMSO-*d*_6_) *δ* 11.52 (s, 1H), 8.33 (t, 1H, *J* = 5.3 Hz), 8.17 (d, 1H, *J* = 5.2 Hz), 8.09 (d, 1H, *J* = 8.6 Hz), 7.85 (d, 1H, *J* = 5.1 Hz), 7.43–7.36 (m, 4H), 7.34–7.30 (m, 1H), 7.26 (s, 1H), 7.07 (s, 1H), 6.90 (d, 1H, *J* = 8.4 Hz), 4.22 (t, 2H, *J* = 5.2 Hz), 3.63 (q, *J* = 5.4 Hz, 2H), 2.75 (s, 3H), 2.04 (s, 3H); ^13^C NMR (DMSO-*d*_6_) *δ* 169.15, 159.42, 142.12, 141.01, 137.10, 136.02, 134.52, 132.50, 132.28, 129.21, 128.40, 127.70, 127.53, 122.79, 114.93, 112.08, 109.56, 95.47, 66.28, 38.96, 20.03, 14.29; ESI-MS: *m/z* 386.3 (M + 1)^+^; Anal. Calcd. for C_24_H_23_N_3_O_2_: C, 74.78; H, 6.01; N, 10.90, found: C, 74.99; H, 6.32; N, 10.73.

### 3.2. In Vitro Drug Sensitivity Assay against Erythrocytic Stages of P. falciparum

The antiplasmodial activity of harmicines **5** and **6** was evaluated against two laboratory *P. falciparum* strains (3D7 – CQ-sensitive, and Dd2 – CQ-resistant), as previously described, using the histidine-rich protein 2 (HRP2) assay [23,24]. Briefly, 96-well plates were pre-coated with the tested compounds in a three-fold dilution before ring stage parasites were added in complete culture medium at a hematocrit of 1.5% and a parasitemia of 0.05%. After three days of incubation at 37 °C, 5% CO_2_ and 5% oxygen, plates were frozen until analyzed by HRP2-ELISA. All compounds were evaluated in duplicate in at least two independent experiments. The IC_50_ was determined by nonlinear regression analysis of log concentration–response curves using the drc package v0.9.0 of R v2.6.1 [22].

### 3.3. In Vitro Activity against P. berghei Hepatic Stages

The in vitro activity of harmicines **5** and **6** against the liver stages of *P. berghei* infection was assessed as previously described [25,26]. Briefly, Huh7 cells were routinely cultured in 1640 Roswell Park Memorial Institute (RPMI) medium supplemented with 10% (*v/v*) fetal bovine serum, 1% (*v/v*) glutamine, 1% (*v*/*v*) penicillin/streptomycin, 1% non-essential amino acids, and 10 mM 2-(4-(2-hydroxyethyl)piperazin-1-yl)ethanesulfonic acid (HEPES). For drug screening experiments, Huh7 cells were seeded at 1 × 10^4^ cell/well of a 96-well plate and incubated overnight at 37 °C with 5% CO_2_. Stock solutions of test compounds (10 mM) were prepared in DMSO and were serially diluted in infection medium, i.e., culture medium supplemented with gentamicin (50 μg/mL) and amphotericin B (0.8 μg/mL), in order to obtain the test concentrations. On the day of the infection, the culture medium was replaced by serial dilutions of test compounds and incubated for 1 h at 37 °C with 5% CO_2_. Next, 1 × 10^4^ firefly luciferase-expressing *P. berghei* sporozoites, freshly isolated from the salivary glands of female infected *Anopheles stephensi* mosquitoes, were added to the cultures, plates were centrifuged at 1800× *g* for 5 min at room temperature and incubated at 37 °C with 5% CO_2_. To assess the effect of each compound concentration on cell viability, cultures were incubated with Alamar Blue (Invitrogen, Waltham, MA, USA) at 46 h post infection (hpi), according to the manufacturer’s recommendations. The parasite load was then assessed by a bioluminescence assay (Biotium, Fremont, CA, USA), using a multi-plate reader, Infinite M200 (Tecan, Männedorf, Switzerland). Nonlinear regression analysis was employed to fit the normalized results of the dose–response curves, and IC_50_ values were determined using GraphPad Prism 6.0 (GraphPad software, La Jolla, CA, USA).

### 3.4. In Vitro Cytotoxicity Assay

Cytotoxicity against a human cell line (HepG2) was evaluated using the neutral red assay [27]. In brief, human cells were seeded to a 96 well plate in complete culture medium, before on the following day a serial dilution of the respective compound was added. After one day of incubation, cytotoxicity was evaluated by the addition of Neutral Red, subsequent lysis of cells and the measurement of absorbance in a plate reader. The IC_50_ was determined as for the in vitro drug assay against *P. falciparum.* To assess the safety of a compound, SI was calculated as the fractional ratio between the IC_50_ values for HepG2 and the *P. falciparum* 3D7 strain.

### 3.5. Molecular Dynamics Simulations

The starting point of our molecular dynamics simulations was a *Pf*Hsp90 N-terminal domain structure obtained by X-ray crystallography from the Protein Data Bank (accession code 3K60). Ligands (ADP and SO_4_^2−^) were removed from the model and selected compounds were placed in the ATP binding pocket, including harmine as a reference. Original crystal waters were removed from the structure so that water molecules from the bulk solvent could diffuse into the protein during equilibration and production MD runs. In order to parameterize the investigated ligands, geometry optimization and RESP charge calculations were performed using the Gaussian 16 program [31] at the HF/6–31G(d) level to be consistent with the employed GAFF force field, while the *Pf*Hsp90 protein was modeled using the AMBER ff14SB force field. Such protein complexes were solvated in a truncated octahedral box of TIP3P water molecules spanning a 10-Å-thick buffer, neutralized by Na^+^ ions and submitted to geometry optimization in the AMBER 16 program [32] by employing periodic boundary conditions in all directions. Optimized systems were gradually heated from 0 to 300 K and equilibrated during 30 ps using NVT conditions, followed by productive and unconstrained MD simulations of 300 ns by employing a time step of 2 fs at a constant pressure (1 atm) and temperature (300 K), with the latter held constant using a Langevin thermostat with a collision frequency of 1 ps^−1^. Bonds involving hydrogen atoms were constrained using the SHAKE algorithm [33] while the long-range electrostatic interactions were calculated employing the Particle Mesh Ewald method [34]. The nonbonded interactions were truncated at 10.0 Å.

The binding free energies, Δ*G*_BIND_, of each ligand within the *Pf*Hsp90 ATP binding site were calculated using the established MM-GBSA protocol [35,36] available in AmberTools16 [32], and in line with our earlier reports [15,37,38]. MM-GBSA is a widely used method for binding free energy calculations from snapshots of MD trajectory with an estimated standard error of 1–3 kcal mol^−1^ [36]. For that purpose, 1000 snapshots collected from the last 30 ns of the corresponding MD trajectories were utilized. The calculated MM-GBSA binding free energies were decomposed into specific residue contributions on a per-residue basis according to the established procedure [39,40]. This protocol calculates contributions to Δ*G*_BIND_ arising from each amino acid residue and identifies the nature of the energy change in terms of interaction and solvation energies or entropic contributions.

## 4. Conclusions

In this paper, we have presented a synthesis of the amide-type harmicines, which is simpler and more efficient than the synthesis of the triazole-type harmicines. This, in turn, offers significant advantages in terms of the cost and availability of those compounds. We also investigated their biological activities against erythrocytic and hepatic stages of *Plasmodium*, as well as against the HepG2 cell line and performed computational analysis in order to gain more insight into their binding to *Pf*Hsp90. Amide-type harmicines exerted stronger antiplasmodial activities against the erythrocytic stage of infection than their triazole counterparts. At the same time, their activities against hepatic stages of *P. berghei* were not significant. Furthermore, *N*-harmicines displayed favorable selectivity indices. Molecular dynamics simulations indicated that their binding to *Pf*Hsp90 might be crucial for the inhibition of *Plasmodium* development. These results pave the way for the future enrichment of the harmicines library, towards the establishment of a relevant QSAR model and the identification of a lead compound for further development.

## Figures and Tables

**Table 1:** In vitro screening of antiplasmodial activity of amide-type harmicines **5** and **6** against erythrocytic stage of *P. falciparum* (*Pf*3D7 and *Pf*Dd2 strains), cytotoxicity of human liver hepatocellular carcinoma cell line (HepG2) and calculated selectivity indices. Data for harmine and chloroquine are taken from [15].

Compd.	IC_50_^1^ (µM)			SI ^2^
	*Pf*3D7	*Pf*Dd2	HepG2	
**5a**	0.49 ± 0.25 ^3^	1.11 ± 0.15 ^3^	350.31 ± 13.02 ^3^	715
**5b**	0.07 ± 0.03	0.78 ± 0.32	54.11 ± 13.36	773
**5c**	0.07 ± 0.03	0.41 ± 0.01	2.88 ± 0.42	41
**5d**	0.09 ± 0.06	0.33 ± 0.11	20.99 ± 0.88	233
**5e**	0.04 ± 0.02	0.17 ± 0.01	2.91 ± 1.75	73
**5f**	0.26 ± 0.001	0.49 ± 0.24	74.69 ± 7.61	287
**6a**	0.98 ± 0.12	4.7 ± 2.65	12.74 ± 0.66	13
**6b**	2.75 ± 1.6	5.01 ± 0.83	12.86 ± 3.31	5
**6c**	0.37 ± 0.22	0.48 ± 0.28	6.11 ± 2.07	16
**6d**	0.15 ± 0.06	0.69 ± 0.18	7.63 ± 2.47	51
**6e**	0.32 ± 0.03	0.4 ± 0.24	7.72 ± 3.04	24
**6f**	0.21 ± 0.14	1.09 ± 0.49	10.53 ± 0.43	50
**6g**	0.93 ± 0.28	3.92 ± 1.35	16.17 ± 1.86	17
**6h**	6.78 ± 0.72	19.53 ± 11.83	61.28 ± 2.75	9
CQ ^4^	0.003 ± 0.002	0.20 ± 0.10	n.d.	n.d.
Harmine	8.25 ± 2.83	>27.7	> 250	30

^1^ IC_50_, the concentration of the tested compound necessary for 50% growth inhibition. ^2^ SI, selectivity index, ratio between IC_50_ (HepG2) and IC_50_ (*Pf*3D7). ^3^ Results represent mean ± SD, *n* ˃ 2. ^4^ CQ, chloroquine.

**Table 2:** Calculated total binding free energies (Δ*G*_BIND_) from molecular dynamics trajectories using MM-GBSA approach, and their decomposition on a per-residue basis for selected derivatives. ^1^ Data for harmines are taken from [15].

Residue	5a	5d	5e	6a	6d	6e	Harmine
Asn37	−1.82	−1.33	−2.01	−2.09	−1.69	−1.81	0.00
Asp79	1.47	1.36	1.58	1.11	1.30	0.74	0.00
Arg98	−0.05	−0.93	−1.39	−2.00	−2.45	−2.24	0.00
Phe124	−1.44	−1.68	−0.64	−0.58	−0.35	−0.37	0.00
Met84	−2.50	−2.63	−2.70	−2.05	−2.25	−2.02	0.00
Gly83	−0.79	−1.28	−1.63	−1.00	−1.69	−1.56	0.00
Ile82	−0.88	−1.50	−1.51	−1.28	−1.49	−1.43	0.00
Thr171	−1.60	−1.92	−1.51	−0.74	−0.51	−0.53	0.00
Ala41	−1.39	−1.37	−1.44	−1.32	−1.40	−1.27	0.00
Ile173	−1.05	−1.13	−1.18	−0.46	−0.42	−0.32	−0.01
Asn92	−0.96	−0.94	−1.06	−1.64	−1.43	−1.41	0.00
Ala38	−0.90	−0.91	−0.89	−0.56	−0.53	−0.50	0.00
Leu93	−0.63	−0.60	−0.80	−0.46	−0.52	−0.59	0.00
Leu34	−0.68	−0.82	−0.75	−0.16	−0.14	−0.12	0.00
Val136	−0.67	−0.72	−0.64	−0.11	−0.14	−0.12	−0.02
Gly81	−0.33	−0.49	−0.54	−0.37	−0.42	−0.35	0.00
Thr95	0.03	0.03	0.03	0.03	0.04	0.04	0.00
Asp142	0.04	0.03	0.04	0.03	0.03	0.03	0.00
Glu48	0.03	0.05	0.04	0.05	0.06	0.06	0.00
Total Δ*G*_BIND_	−33.9	−38.1	−40.9	−34.6	−35.7	−37.5	−7.5

^1^ Residues are selected to list those identified as belonging to the ATP binding pocket (Asn37, Asp79, Arg98, Phe124) and all of those with contributions higher than −0.50 and lower than 0.03 kcal mol^−1^ to the most potent **5e**. All values are in kcal mol^−1^.

## Supplementary Materials

Table S1: Properties of novel compounds calculated with Chemicalize.org program. The Lipinski’s and Gelovani’s parameters; Table S2: IR, ^1^H and ^13^C NMR spectroscopic data for compounds **1** and **3**; Table S3: IR, ^1^H and ^13^C NMR spectroscopic data for compounds **2** and **4**; Table S4: Analytical and MS data for harmicines **5a–f**; Table S5: IR, ^1^H and ^13^C NMR spectroscopic data for harmicines **5a–f**; Table S6: Analytical and MS data for harmicines **6a–h**; Table S7: IR, ^1^H and ^13^C NMR spectroscopic data for harmicines **6a–h**; spectra of all compounds are available online.

## Abbreviations

ADP	adenosine diphosphate
ATR	attenuated total reflectance
Boc	*tert*-butyloxycarbonyl
CAD	cinnamic acid derivative
DCM	dichloromethane
DIEA	*N*,*N*-diisopropylethylamine
DMF	*N*,*N*-dimethylformamide
ESI	electrospray ionization
GAFF	generalized Amber force field
HATU	1-[bis(dimethylamino)methylene]-1*H*-1,2,3-triazolo[4,5-*b*]pyridinium-3-oxidhexafluorophosphate
HEPES	2-(4-(2-hydroxyethyl)piperazin-1-yl)ethanesulfonic acid
HRP2	histidine-rich protein 2
HepG2	human liver hepatocellular carcinoma cell line
IC_50_	the concentration of the tested compound necessary for 50% growth inhibition
MD	chloroquine-sensitive strain of *P. falciparum*
MM-GBSA	molecular mechanics/generalized born surface area
MW	microwave
*Pf*Dd2	chloroquine-resistant strain of *P. falciparum*
*Pf*Hsp90	*P. falciparum* heat shock protein 90
QSAR	quantitative structure–activity relationship
RESP	restrained electrostatic potential
SI	selectivity index
TIP3P	transferable intermolecular potential with 3 points
TMS	tetramethylsilane
UATR	universal attenuated total reflectance
